# Introduction of the YTE mutation into the non-immunogenic HIV bnAb PGT121 induces anti-drug antibodies in macaques

**DOI:** 10.1371/journal.pone.0212649

**Published:** 2019-02-20

**Authors:** Yvonne J. Rosenberg, George K. Lewis, David C. Montefiori, Celia C. LaBranche, Mark G. Lewis, Lori A. Urban, Jonathan P. Lees, Lingjun Mao, Xiaoming Jiang

**Affiliations:** 1 PlantVax Corporation, Rockville, Maryland United States of America; 2 Institute of Human Virology, University of Maryland School of Medicine, Baltimore, Maryland, United States of America; 3 Department of Surgery, Duke University Medical Center, Durham, North Carolina, United States of America; 4 Bioqual Inc. Rockville, Maryland United States of America; Emory University School of Medicine, UNITED STATES

## Abstract

Recombinant antibodies play increasingly important roles as immunotherapeutic treatments for human cancers as well as inflammatory and infectious diseases and have revolutionized their management. In addition, their therapeutic potential may be enhanced by the introduction of defined mutations in the crystallizable fragment (Fc) domains eg YTE (M252Y/S254T/T256E) and LS (M428L/N434S), as a consequence of increased half-lives and prolonged duration of protection. However, the functional properties of any biologic may be compromised by unanticipated immunogenicity in humans, rendering them ineffective. Several potent broadly neutralizing HIV monoclonal antibodies (bnAbs) have been identified that protect against SHIV challenge in macaque models and reduce HIV viremia in HIV-infected individuals. In the present study, the pharmacokinetics and immunogenicity of one or more 5mg/kg subcutaneous (SC) injections in naïve macaques of the HIV bnAb PGT121 and its PGT121-YTE mutant, both produced in plants, have been compared towards prolonging efficacy. Induction of anti-drug/anti-idiotypic antibodies (ADA, anti-id) has been monitored using both binding ELISAs and more functional inhibition of virus neutralization (ID50) assays. Timing of the anti-Id responses and their impact on pharmacokinetic profiles (clearance) and efficacy (protection) have also been assessed. The results indicate that ADA induction in naïve macaques may result both from injection of the previously non-immunogenic PGT121 into pre-primed animals and also by the introduction of the YTE mutation. Binding ADA antibody levels, induced in 7/10 macaques within two weeks of a first or second PGT121-YTE injection, were closely associated with both reduced pharmacokinetic profiles and loss of protection. However no correlation was observed with inhibitory ADA activity. These studies provide insights into both the structural features of bnAb and the immune status of the host which may contribute to the development of ADA in macaques and describe possible YTE-mediated changes in structure/orientation of HIV bnAbs that trigger such responses.

## Introduction

The success of recent single B cell cloning from HIV infected individuals has resulted in a new generation of broad and highly potent HIV monoclonal antibodies [1,2] that can both prevent infection in non-human primates against SHIV challenge [3–5] and suppress viremia in NHP [6,7], humanized mice [8,9] and humans [10,11].

Different bnAbs are known to target non-overlapping epitopes on the HIV envelope [12] including the membrane proximal region [13,14], the apical V1/V2 loops [2,15], the base of the V3 loop and associated glycans [16,17], the CD4 binding site [18,19], and epitopes that span gp120 and gp41 [20]. PGT121 is one of the most potent neutralizing bnAbs targeting both the oligo-mannose glycan at N332 as well as the conserved 324GD/NIR327 peptide motif at the base of the gp120 V3-loop [21,22] a consequence of having a long CDRH3s that can penetrate the glycan shield. This antibody differs from its family member 10–1074 which interacts more strongly with glycans at positions N137, N156, and N301, and is less likely to be dependent on the N332 glycan [23]. In previous studies, plant-derived PGT12 was unusual in that it was shown to be consistently non-immunogenic in the naïve macaques used [24]. Thus, subcutaneous administration of PGT121by itself protected the same macaques following two consecutive injections and challenges given months apart [5]. This lack of immunogenicity of PGT121 (as well as b12) in macaques is in contrast to many other HIV bnAbs tested and is likely a result of the low amino acid mutation rates in PGT121 (23%) and b12 (20%) and not reflective of the higher 34% and 28% mutation rate in the PGT121 VH and VL at the DNA level [25].

To further extend the duration of protection and viral suppression, several defined mutations have also been introduced into the crystallizable fragment (Fc) domains of immunotherapeutic mAbs which result in increased half-lives and/or effector function eg ADCC [26,27]. In this context, both the YTE (M252Y/S254T/T256E) and LS M428L/N434S) mutations located at the CH2-CH3 interface in the Fc domain have been shown to increase the binding affinity of the antibody Fc at pH 6.0 to the MHC Class I neonatal FcR (FcRn), located primarily in the acidic endosomes of endothelial and haematopoietic cells, thereby permitting more efficient recycling of administered IgG1 antibody and longer retention in the plasma [26–29]. The increased FcRn binding at pH 6.0 by a YTE triple-mutant mAb is mediated by the creation of one additional salt bridge between Glu 256 (E) of Fc-YTE and Gln 2(Q) of the b2-microglobulin chain of FcRn compared to the original IgG1 Fc structure [28]. Thus, introduction of the YTE mutation into the protective anti-respiratory syncytial virus (RSV) antibody motavizumab (MEDI8897, a follow on candidate to Synagis) resulted in ten-fold higher FcRn binding, with 4-fold increases in circulatory retention time and lung bioavailability in cynomologus monkeys [29] and has been shown to be well tolerated and extended the half-life up to 100 days in adult humans and pre-term infants [30,31].

Similarly, YTE and LS substitutions of the humanized anti-VEGF IgG1 antibodies bevacizumab and cetuximab lead to increased FCRn binding at pH 6.0 and enhancement of half-life (3.2-fold and 3.1-fold respectively) as well as improved antitumor activity [32] in cynomolgus macaques. More recently, the LS mutation has also been shown to increase half-lives and prolong duration of protection of anti-HIV bnAbs in macaques but in some cases with variable pharmacokinetics [33, 34].

However, while high potency, wide breadth of coverage and increased half-lives are early prerequisites for their therapeutic applications, challenges to the use of HIV bnAbs as treatments include (i) emergence of escape variants within weeks following infusion which may be more frequent in the V3/N332 bnAbs e.g 10–1074, compared to CD4 binding bnAbs [10] and (ii) the development of anti-drug (ADA) antibody which could rapidly negate any other therapeutic advantages associated with potency [24,33,35]. Approval of biological products is determined by the extent of their immunogenicity and is reflected in the prescribing information at Drugs@FDA website under Section 6 Adverse Reactions and Section 12.3 Pharmacokinetics. Assessment of immunogenicity in clinical trials and terminology recommendations have been described in reports by Wang et al [36] and Shankar et al. [37] and discussed later.

Since YTE mutants were the first used in clinical trials [30], the current macaque study compared the pharmacokinetics and immunogenicity of unmodified PGT121 with its YTE (M252Y/T254S/T256E) mutant form to examine the extent to which circulatory residence time and thus protection can be extended by this mutation.

The results indicate that YTE-substituted HIV bnAbs unexpectedly exhibit increased immunogenicity and accelerated circulatory clearance, rather than enhanced plasma stability, suggesting a role for the CH2-CH3 interface in the Fc domain. To our knowledge, this work is the first to demonstrate introduction of the YTE mutations, where increased flexibility and decreased conformational stability of the adjacent CH2 segment may also result in reorientation of the antibody an exposure of potentially novel epitopes.

The timing and clinical relevance of PGT121-YTE binding versus inhibitory anti-drug antibodies has been assessed by examining the association between immunogenicity, pharmacokinetics and efficacy following SHIV SF162P3 challenge and indicate the importance of clearance profiles in predicting ADA responses.

## Methods and materials

### Antibody production in plants

Monoclonal antibody PGT121 was produced by *Agrobacterium*-mediated transient gene expression in *N*. *benthamiana* as described previously [38,39]. Synthetic codon optimized variable domains were flanked by type-IIs restriction sites and cloned into pTRAk plant expression vectors carrying the kappa constant domain as well as the YTE substituted (M252Y/S254T/T256E) IgG1 H constant domain. The originally published antibody amino acid sequences were used unless indicated otherwise. Antibodies were produced by co-infiltrating 6-week old plants with recombinant *Agrobacteria* suspensions individually carrying the pTRAk based heavy and light chain expression plasmids and the pBIN based p19 silencing suppressor from tomato bushy stunt virus. After 10–12 days, infiltrated leaves were harvested and soluble proteins were extracted and purified by protein-A (Genscript, NJ) and MEP HyperCel^TM^ mixed-mode chromatography (Pall Corporation, France) producing 600–1,500 mg/kg of leaf biomass depending on the antibody. YTE mutants were also purified by Protein A indicating that mutatations did not interfere with the Protein A binding site. The VRC01^**N92T**^, PGDM1400-YTE and 3BNC117-YTE, N6-YTE expressed in plants using the same method as PGT121, were also used in Neutralization and ELISA assays. Purified bnAbs can be stored at 4°C or frozen until use.

### Non-human primates

Rhesus macaques (*Macaca mulatta*) (3-6kg) were housed at BIOQUAL's housing facilities in Rockville, MD. Care and husbandry of all non-human primates were provided in compliance with federal laws and guidelines as well as in accordance with recommendations provided in the NIH guide and other accepted standards of laboratory animal care and use. BIOQUAL is accredited by the Association for the Assessment and Accreditation of Laboratory Animal Care, (AAALAC file #624) and holds an Assurance on file with the National Institute of Health, Office for Protection of Research Risks as required by the US Public Health Service Policy on Humane Care and Use of Laboratory Animals. The PHS Animal Welfare Assurance File Number is #A-3086–01. Animals were sedated with ketamine or telazol for all technical procedures. Ketamine was given IM in amounts necessary for short-term procedures such as blood drawing.

Animals were housed at BIOQUAL, Inc. MD, in accordance with the recommend-dations of the Association for Assessment and Accreditation of Laboratory Animal Care International Standards and with the recommendations in the NIH Guide for the Care and Use of Laboratory Animals of the United States. The Institutional Animal Use and Care Committee of BIOQUAL approved these experiments (#18-058P Plantvax Renewal (for IACUC #15–059). When immobilization was necessary, the animals were sedated intramuscularly with 10 mg/kg of Ketamine HCl (Parke-Davis, Morris Plains N.J.) before any direct handling or procedures. All efforts were made to minimize suffering. Details of animal welfare and steps taken to ameliorate suffering were in accordance with the recommendations of the Weatherall report, “The use of non-human primates (NHP) in research”. Animals were housed in an air-conditioned facility with an ambient temperature of 21–25°C, a relative humidity of 40%–60% and a 12 h light/dark cycle. Animals were socially housed when possible or individually housed if no compatible pairing could be found. The animals were housed in suspended stainless steel wire-bottomed 6 sq ft cages and provided with a commercial primate diet and fresh fruit and vegetables twice daily with water freely available at all times. Social housing, toys, foraging equipment and mirrors were provided. Animals were monitored at least twice daily for behavior, food intake, activity, and overall health by trained technicians. No macaques were euthanized and all animals were returned to the colony.

### Pharmacokinetic and immunogenicity studies

Pharmacokinetic and immunogenicity studies of the plant produced HIV mAbs PGT121 and PGT121-YTE were performed at Bioqual using 3–6 kg naïve female Indian rhesus macaques (*Macaca mulatta*). To assess plasma retention of each bnAb, macaques were injected once or twice with 5 mg/kg of filtered, room-temperature PGT121 and PGT121-YTE in 1–1.5 ml of PBS (pH 7.0) SC in a single injection in the middle of the back in addition to IM Benadryl 30 mins prior to and immediately post injection. Animals were bled (0.5 ml) from the femoral artery at time zero and for 2–3 weeks at the times indicated. In one study, a second injection was administered 6 weeks later. Studies using each bnAb were repeated several times. Usually if ADA was produced after the first injection, no second injection was given. Plasma or serum samples were tested for both levels of circulating mAb measured by ELISA or by neutralizing antibody activity (ID50) (Duke University). The induction of anti-human bnAb antibody (ADA/ anti-Id) was assessed by ELISA and inhibition of neutralization assays (ID50, Duke University) as described previously (24). Four pharmacokinetic parameters, based on the time course of bnAb clearance in the blood were examined: Mean Retention time (MRT), Maximum concentration (Cmax), half-life (T1/2) and area under curve (AUC). An Excel-based PK Solutions 2.0 program (Summit Research Services, CO) for non-compartmentalized analysis of pharmacokinetic data, was used to analyze data.

### Neutralization assays

Neutralizing antibody assays were performed in TZM-bl cells as previously described [40] with purified bnAbs and also with plasma samples collected from macaques at different times following SC injection of the bnAbs. Purified recombinant antibodies were tested starting at 50μg/ml with serial 3-fold dilutions. Plasma (both heat-inactivated and non-heat-inactivated) was tested starting at a 1:20 dilution. Diluted test samples were pre-incubated with pseudovirus (~150,000 relative light unit equivalents) for 1 hr at 37°C before addition of cells. Following 48 hr incubation, cells were lysed and luciferase (Luc) reporter gene activity determined using a microtiter plate luminometer and BriteLite Plus Reagent (Perkin Elmer). Neutralization titers are the sample dilution (for plasma) or antibody concentration (for purified mAb) at which relative luminescence units (RLU) were reduced by 50% compared to RLU in virus control wells after subtraction of background RLU in cell control wells. It should be noted that introduction of the YTE mutation did not change the IC50 of the PGT121 bnAb.

Inhibition of neutralization assays to detect inhibitory anti-id antibody were also performed in TZM-bl cells [24]. Initially, a concentration of mAb that inhibited the target virus at 50–80% was pre-incubated with or without serial dilutions of monkey plasma samples for 1 hr at 37°C prior to adding virus. After an additional 1 hour incubation of mAb/serum/virus, cells were added and the assay was continued according to the standard protocol. The ‘No Serum’ control indicates the level of mAb inhibition of virus. Deviations from this line indicates interference from the plasma sample with neutral-ization of the mAb.

### ELISA

Two types of ELISAs at RT were used to determine the pharmacokinetics and immunogenicity of the administered bnAbs. Firstly, to monitor the rates of clearance of the circulating bnAbs, 96-well MaxiSorp plates (Nunc) were coated with purified plant-derived high mannose 89.6P gp140-KDEL (1μg/ml) for 2–4 hr. In some case e.g. detection of PGT121 levels, plates were coated with CHO-derived monomeric HIV BaL-gp120 (NIH HIV Reagent Program). Plates were washed 3 times with PBST, blocked with 5% (w/v) milk in PBST for 2hr, washed 3 times, incubated for 2 hr with monkey plasma or serum samples at 1/500 dilution, washed 3 times and incubated with a 1/5,000 dilution of peroxidase labeled goat anti-human IgG (Fc) (A0170, Sigma) for 2hr, washed 5 times and developed with KPL SureBlue Reserve TMB Microwell Peroxidase Substrate System (5120–0082, SeraCare MA). Reactions were stopped with 0.5 N H_2_SO_4_, and endpoints were determined at 450 nm using the SPECTRA max PLUS plate reader (Molecular Devices). Due to the variability in the background of individual macaques, the initial prebleed OD450 values were subtracted.

Secondly, to monitor the presence of a macaque antibody response against the injected human HIV bnAbs, plates were coated with the purified plant- or CHO cell-derived target antibodies at 1.2 μg/ml for 2–4 hr [24]. Following incubation, wells were blocked, washed and incubated for 2 hr with monkey plasma or serum samples at 1/500 dilutions followed by a third 2 hr incubation with 1/4,000 of mouse anti-macaque IgG (1B3-HRP, Nonhuman Primate Reagent Resource) and developed as above. In some anti-id ELISAs both unmodified and YTE mutant forms of PGT121, 3BNC117 and PGDM1400 were used to coat plates.

### Protection studies

In this study, macaques were injected SC with 5 mg/kg of PGT12-YTE, 9 and 13 days prior to intravaginal challenge with a high dose (1700 TCID) of SHIV SF162P3 that has been shown to infect most control animals after a single challenge. For intravaginal challenge, anesthetized macaques were administered SHIV SF162P3 using a non-leuer-lock syringe inserted ~2 cm into the vaginal vault. The potency of the plant-derived PGT121 against the rhesus (R157) PBMC-derived SF162P3 stock used for challenge was 0.08 ug/ml; similar to the IC50 of CHO-derived PGT121 (0.15 ug/ml). Protection was assessed using a viral RNA assay as described [41]. Fisher’s exact test was performed using the R statistical package [42].

## Results

### Induction of anti-PGT-121 antibody in protected macaques

**Fig 1:**
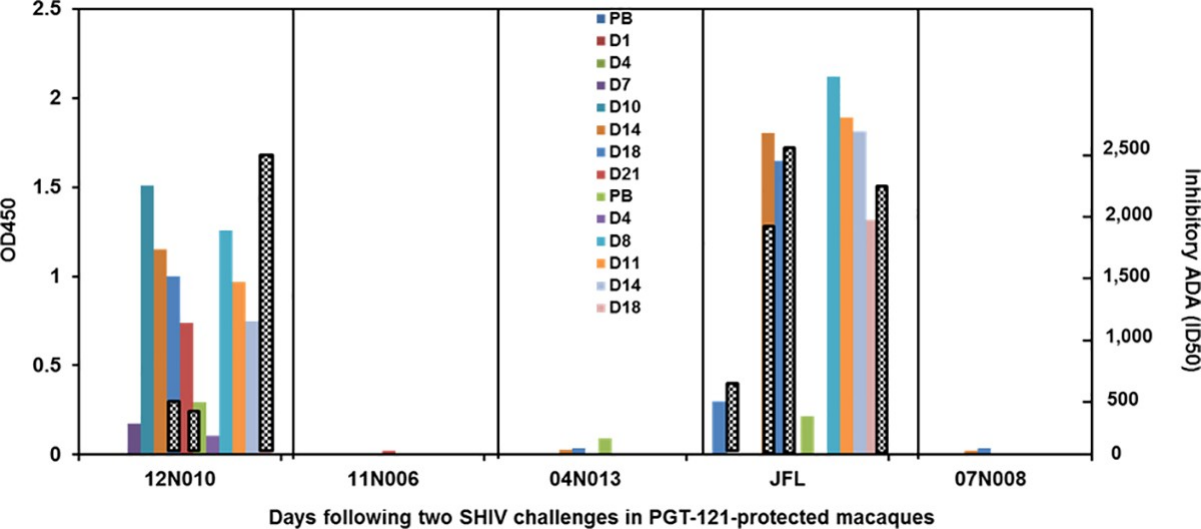
A comparison of binding and inhibitory anti-PGT121 antibodies in sera of protected macaques injected twice with PGT121 (5mg/kg) and challenged twice with SF162P3 [5]. The coloured bars depict binding ADA at OD450 (left axis) at different times after challenge. Black and white bars represent inhibitory ADA (ID50, right axis). PB = prebleed before first and second challenges.

In an earlier study [5], six naïve macaques were administered 5 mg/kg PGT121 SC 24 hr prior to challenge with SF162P3 resulting in protection in all animals. Two months later, these same macaques were challenged with SF162P3 and injected SC with PGT121 30–60 mins later. In the latter case 5/6 macaques were protected. To confirm that a lack of immunogenicity of PGT121 contributed to this protection these five macaques were assessed for the presence of specific anti-PGT121 antibody. As shown in Fig 1, while three (#11N006, #04N013, #07N008) of the five protected macaques did not produce anti-PGT121 antibodies, consistent with previous findings, two macaques (#12N010 and #JFL) surprisingly made high levels of PGT121-binding antibodies (colored bars) detected by ELISA at 7–10 days following both the first and second injections, suggesting that these ADA responses represented a boosting of pre-primed animals. Interestingly, anti-PGT121 antibodies capable of inhibiting PGT121 neutralization of SHIVSF162P3 (black and white bars) demonstrated a different pattern from binding ADA. Thus, inhibitory antibodies in macaque #12N010 were undectable at D0 and reached only low levels of 1/450 (D18) and 1/340 (D21) after the first injection whilst macaque #JFL had preexisting anti-PGT121 antibody (1/625) at the time of the first injection and reached ID50 titers of 1/2,500 in macaques by D14. After the second injection, higher inhibitory titers of 1/2,500, comparable to binding antibody levels were present after second injections in both macaques.

**Fig 2:**
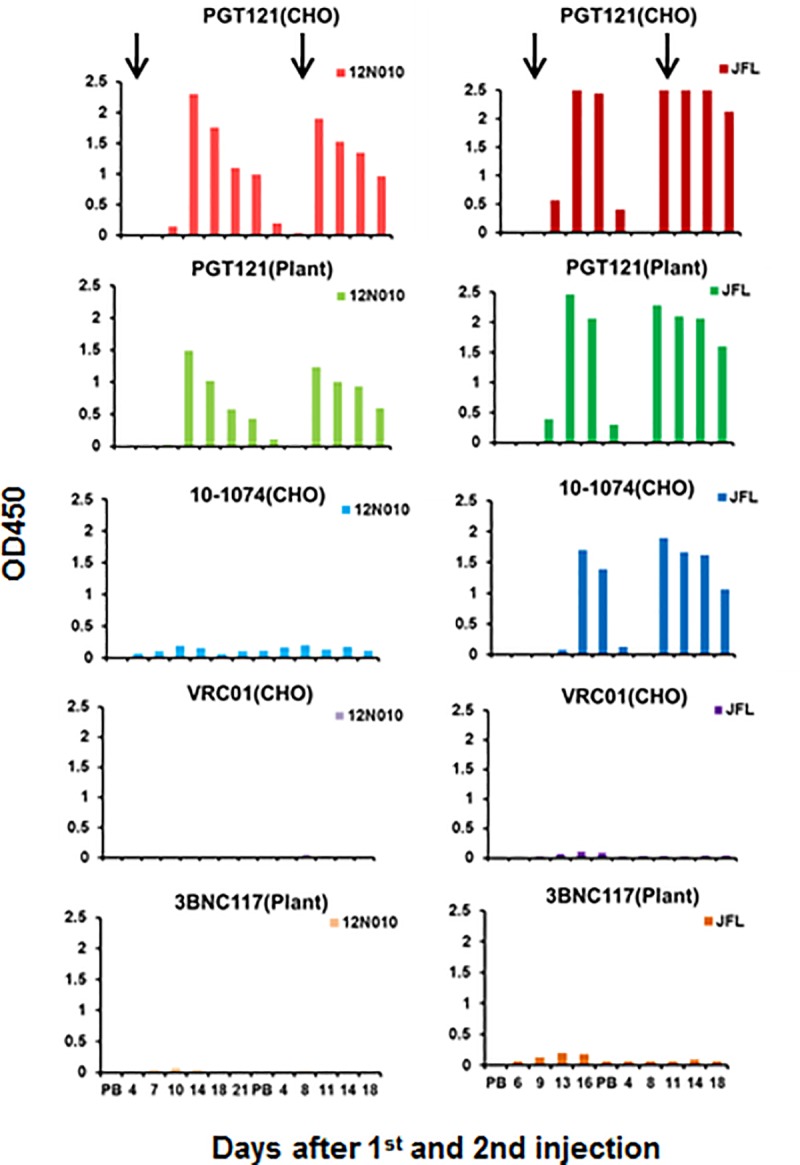
Reactivity of anti-PGT121 antibodies in sera from protected macaques against several HIV bnAbs measured by ELISA. Macaques #12N010 (left) and #JFL (right) were injected twice SC with 5mg/kg PGT121 eight weeks apart and challenged with SHIV SF162P3 24 hr after the first PGT121 injection and one hr before the second PGT121 injection. Sera collected for 2–3 weeks following each challenge was tested for binding against PGT121 (produced in both plants and CHO), 10–1074 (CHO), VRC01 (CHO) and 3BNC117 (plant).

To assess specificity and reactivity of the anti-PGT121 response, sera from macaques #12N010 and #JFL were tested against a panel of different HIV bnAbs using both an ELISA binding assay and neutralization inhibition assays. The binding data in Fig 2 demonstrates the anti-idiotypic (anti-id) nature of these antibodies that sera from both macaques bound well to PGT121, but exhibited no cross-reactivity against other bnAbs eg VRC01 and 3BNC117. The only cross-reactivity was sera from #JFL which also bound well to 10–1074 which shares the same B cell precursor as PGT121 (90% identity) [11,16]. It should be noted that the binding of anti-id to plant-derived PGT121 and CHO-derived PGT121 was similar, indicating that plant contaminants were not responsible for the binding. Specificity was similarly demonstrated in the anti-id inhibition assay in that the anti-PGT121 antibodies did not inhibit neutralization of VRC01 at any time.

### Pharmacokinetics and immunogenicity of PGT121-YTE in macaques

**Fig 3:**
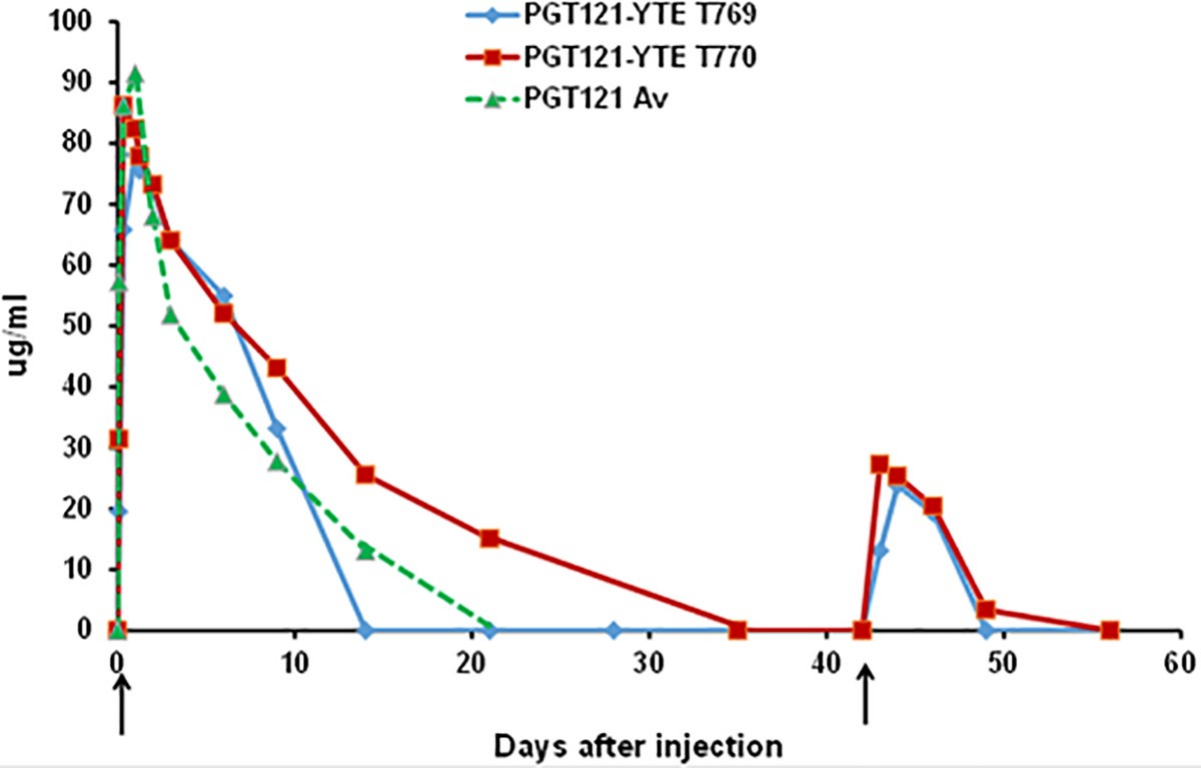
Circulatory clearance profiles of PGTY121-YTE in macaques at different times after two SC injections as measured by ELISA. Two macaques #T769 (blue), T770 (red) received two 5mg/kg injections SC six weeks apart (arrows). Animals were bled at the days indicated and assessed for serum PGT121 levels by ELISA. The green line is an average of two macaques after a single injection of unmodified PGT121 (24).

Interaction between a therapeutic antibody and the FcRn is one of the critical factors in determining half-life and therefore efficacy. In order to extend the circulatory retention of SC-administered PGT121, the triple YTE mutant form (M252Y/S254T/T256E) was produced in plants and analysed in macaques for pharmacokinetics and immunogenicity following SC administration of 5mg/kg. In the first proof-of-concept study, two monkeys received two SC injections of PGT121-YTE 6 weeks apart and their serum levels were monitored using a binding ELISA assay. Fig 3 indicates that at the early day 7 time point following the first injection, both monkeys had a predictable 50% higher PGT121-YTE activity in the circulation compared to the unmodified PGT121 (green line). However, in contrast to macaque #T770 in which PGT121-YTE remained in the circulation until ~35 days representing a modest 2-fold extension compared to unmodified PGT121, the levels in monkey (#T769) dramatically fell to background by ~day 12. In both monkeys, the systemic levels were greatly reduced following a second injection (D43 arrow). The PK parameters for macaques #T770 vs #T769 were Cmax: 86.1 vs 78.1 ug/ml, T1/2: 94 vs 46 hr, AUC¥: 21,637 vs 14,305 ug-hr/ml, MRT: 199 vs 112 hr. PK profiles measured by TMZ-bl neutralization (ID50) were identical to ELISA profiles after the second injection.

**Fig 4:**
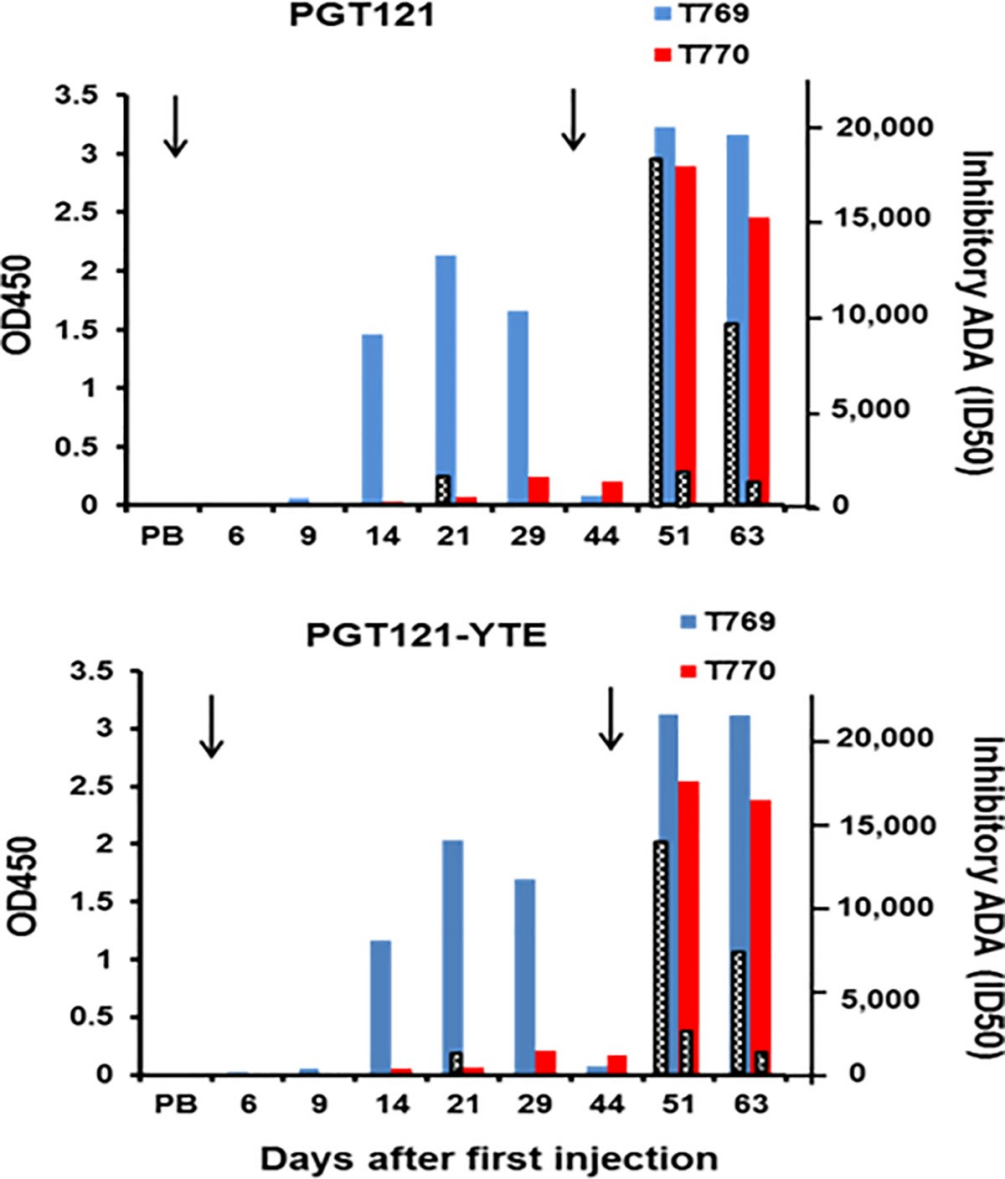
Induction of PGT121 anti-id antibodies in macaques #T769 and T770 injected twice with PGT121-YTE. Macaques #T769 (blue bars) and # T770 (red bars) from Fig 3 were injected SC with 5mg/kg of PGT121-YTE at days 0 and 43 (arrows) and bled at the days indicated. Binding ADA (blue and red bars) were assessed by ELISA using plates coated with unmodified PGT121 (top) and PGT121-YTE (bottom). Inhibitory ADA (ID50) (black and white bars) were also assessed using PGT121 and PGT121-YTE.

This rapid decline of serum PGT121-YTE in #T769 after the first injection and the greatly reduced Cmax after the second correlated with high levels of binding ADA by day 14 after the first injection day 8 after the second (Fig 4, blue bars). By comparison, inhibitory ADA levels in this macaque were very low with ID50 values of 1/144 at day 14 and 1/660 at day 21 (black bars) following the first injection and dramatic increases to 1/17,579 at day 7 and 1/8,768 at day 20 after the second injection. No ADA was induced in #T770 after a single administration by either assay explaining its longer serum presence but it did develop high binding titers (red bars) and moderate inhibitory anti-id (1/1183) by day 8 after the second injection (day 51 in Fig 4), highlighting again the differences between levels of binding and inhibitory ADA in the same samples.

**Fig 5:**
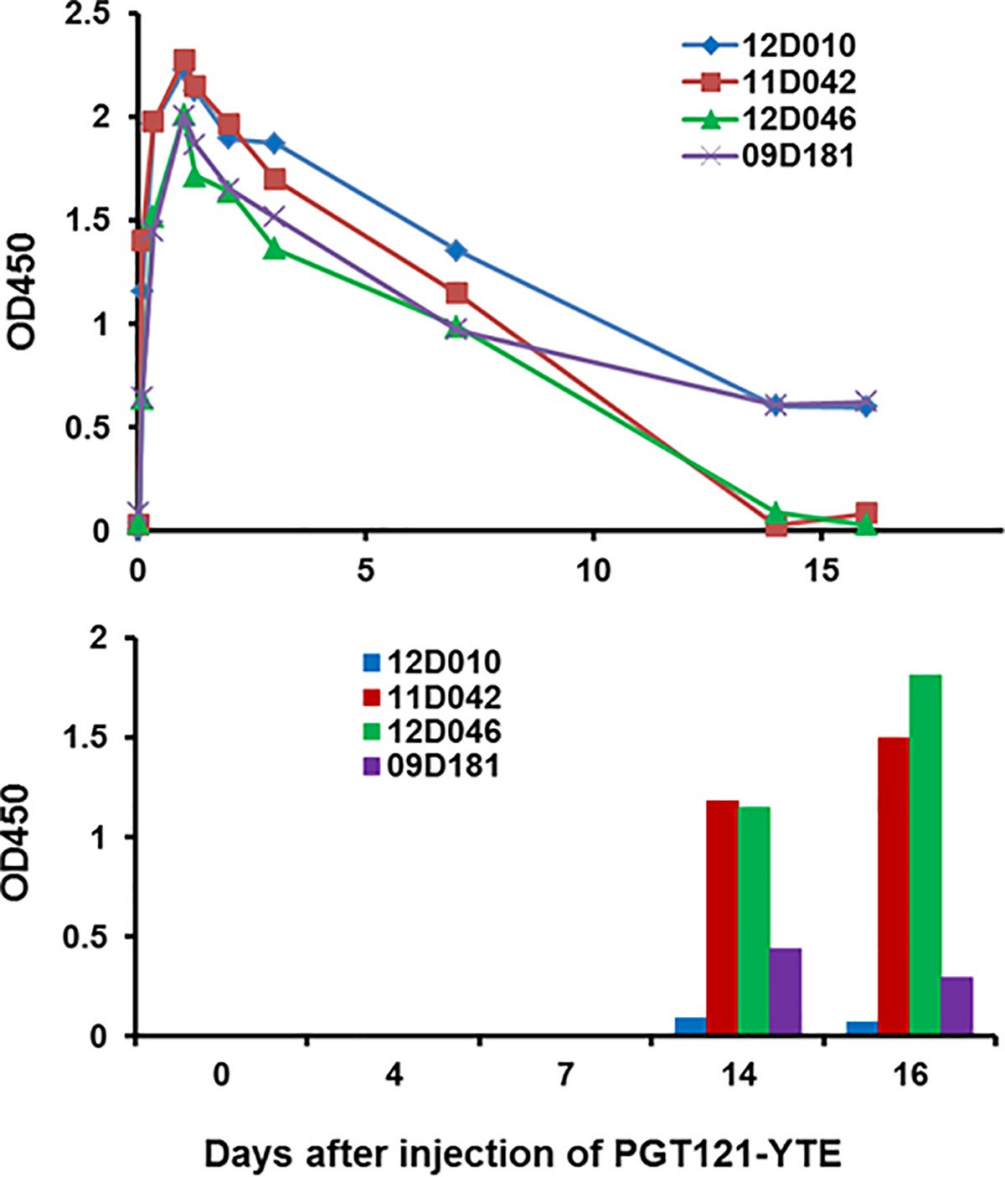
Association in time between the clearance of circulating PGT121-YTE and the induction of binding anti-PGT121-YTE after a single injection. Four macaques received a SC injection of 5mg/kg and were bled and assayed for circulating PGT121-YTE levels on the days indicated (top) and for ADA at days 0,7,14 and 16 (bottom). Macaques #12D010 and #09D181 made no/low anti-id (bars) and had longer PGT121-YTE plasma retention while #11D0924 and #12D046 made anti-id and were thus eliminated from the blood more rapidly.

The results of the early 2-macaque PGT121-YTE study indicated that the YTE mutation may moderately (x2) extend plasma availability following a SC injection but surprisingly leads to a potent immune response after one or two injections of a usually non-immunogenic antibody. Thus in a second study, another four naïve macaques were administered the same dose of PGT121-YTE (5mg/kg) SC and monitored for anti-id using each assay. Fig 5 indicates that a single injection with the PGT121-YTE resulted in binding ADA production in 3/4 naïve monkeys by day 14. Once again, the PK profiles corresponded with the presence of binding ADA antibody in that PGT121-YTE was present in the plasma beyond day 16 in the two monkeys with no/low anti-Id (#12D010, #09D181), but was eliminated between days 7–15 in the two macaques (#12D046 and #11D042) that developed the highest ADA titers. The PK parameters are shown in Table 1. The presence of ADA in the latter monkeys was also detected in a neutralization inhibition assay with low ID50s of 1/171 and 1/156 respectively at day16 after injection

**Table 1:** Pharmacokinetic parameters in four macaques following a single SC injection with 5mg/kg PGT121-YTE.

PK parameter	12D010	09D181	12D046	11D042
T1/2 (hr)	139	177	55	35
Cmax (μg/ml)	131	108	112	133
AUC (μg-hr/ml)	27,443	18,570	13,493	18,499
MRT (hr)	169	211	92	90

### Cross reactivity of anti-PGT121-YTE antibody with other YTE mutants

**Fig 6:**
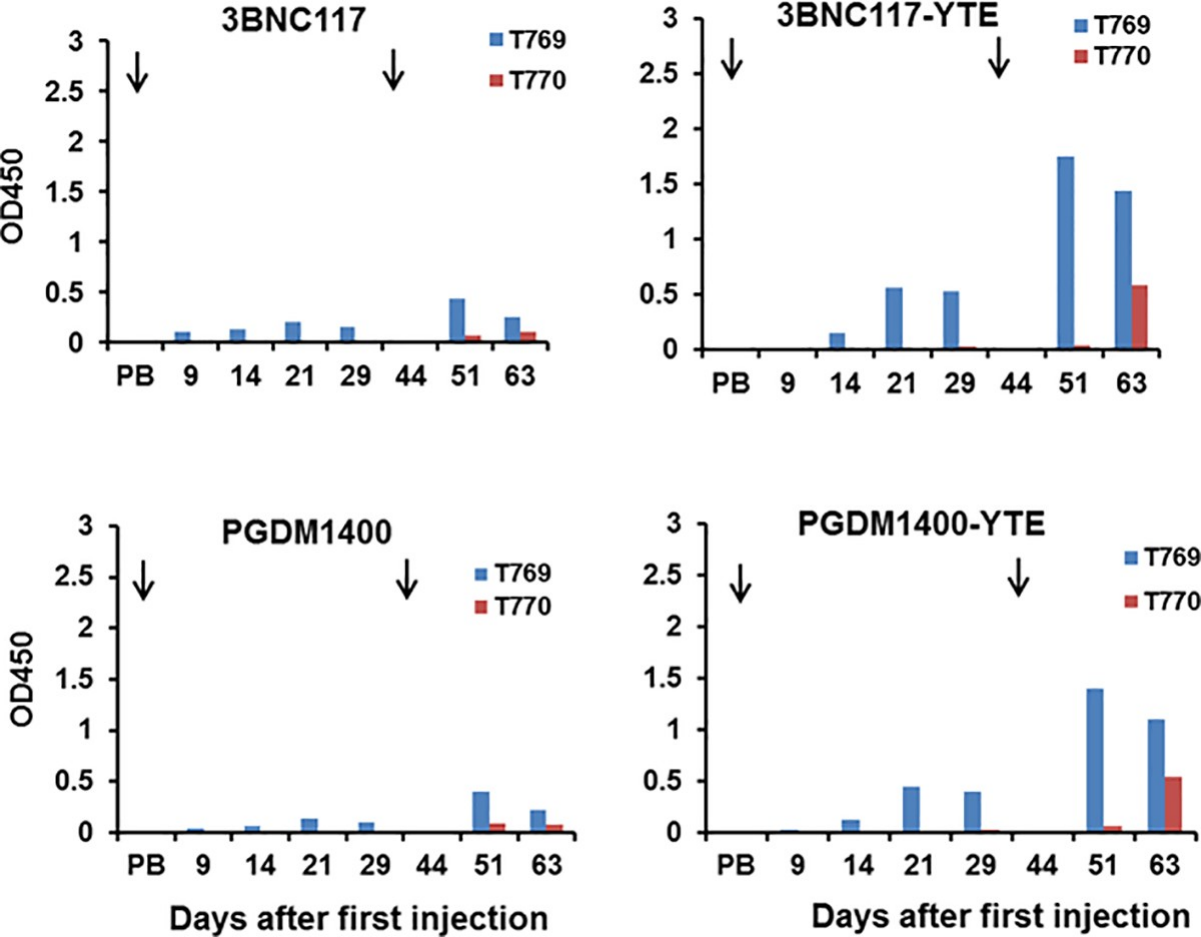
Sera from macaques injected with PGT121-YTE cross-react with PGDM1400-YTE and 3BCN117-YTE mutants. Two monkeys were each injected twice with PGT121-YTE (arrows) and bled at the times indicated. Sera containing anti-PGT121 anti-id antibody (Fig 4) were then tested for binding against unmodified 3BNC117 and PGDM1400 and their YTE mutants.

The above results raised the possibility that the YTE mutation located on the CH2-CH3 margin of the Fc domain, may have affected CH2 mobility and in doing so created new epitopes by altering antibody orientation/conformation; thereby conferring unanticipated immunogenicity on the molecules. Since all HIV bnAbs produced at PlantVax share the same human IgG1, any speculated structural alterations due to the YTE mutation may be common to any Fc domain with the same YTE substitution, in addition to possible Fab changes specific for different cognate antibodies. Thus, the ability of anti-id containing sera from macaques administered PGT121-YTE to bind to different HIV bnAbs with the YTE mutation was examined. Fig 6 compares the binding of sera from macaques #T769 and #T770 injected with PGT121-YTE to either wild type 3BNC117 and PGDM1400 or their YTE mutant forms (Fig 6). In both cases, a much higher reactivity against the YTE forms versus the unmodified forms suggests that the anti-PGT121-YTE antibodies detected by binding may include those with specificity for “YTE-dependent” modifications in or around the CH2-CH3 interfaces of the Fc domains and Fab regions.

Similarly, in this same study, macaques injected SC twice with either 5 mg/kg of PGDM1400-YTE and 3BNC117-YTE were also shown to produce cross-reactive antibodies that bound to all YTE-mutants tested including PGT121-YTE, PGDM1400-YTE and 3BNC117.

### Binding ADA against PGT121-YTE block protection against SF162P3 SHIV

**Fig 7:**
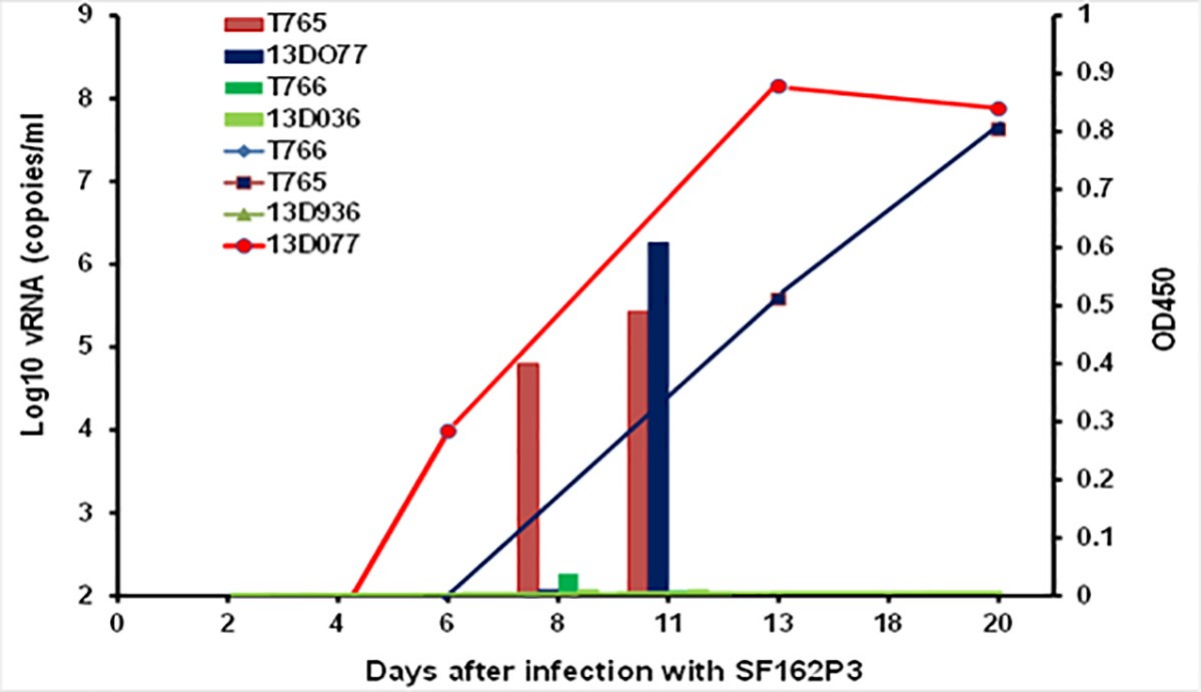
Correlation between the induction of binding anti-PGT121-YTE ADA and viral load. Four macaques received 5mg/kg of PGT121-YTE SC. Two macaques #T766 (dark green) and #13D036 (light green) were challenged with SF162P3 at day 9 and remained uninfected. Two macaques #T765 (red) and #13D077 (blue) were challenged at day 13 and became infected several days apart depending on the onset of ADA induction.

The induction of ADA following administration with a potent mAb may greatly reduce its potential as a therapeutic treatment due to its rapid elimination from the circulation by the anti-Id. In the present studies, anti-PGT121-YTE ADA activity following a single injection of PGT121-YTE was always higher when measured in a binding assay compared to an inhibition assay, raising the question as to which property of the ADA or which assay better predicts elimination of PGT121-YTE to below protective levels. Since, PGT121-YTE ADA were usually detected by ELISA by day 14 after the first injection, a study was performed in which four macaques received 5mg/kg of PGT121-YTE SC and two were challenged at day 9 and two at day 13. The results in Fig 7 indicate that challenge at D9 before ADA was detected by either assay led to protection of macaques #T766 and #13D036, whilst the emergence of ADA (detected by ELISA but not inhibition of neutralization) between D9 and D13 was sufficient to reduce circulating PGT121-YTE to below protective levels leading to infection in macaques # T765 and #13D077 following the D13 challenge. In addition, a close correlation was evident between the timing of ADA response and the SHIV viral load in the two infected macaques. Thus in #T765, the early onset of ADA (red bar) resulted in more rapid removal of PGT121-YTE and early breakthrough of viral replication (red line), whilst in #13D036 the later appearance of ADA (blue bar) permitted sufficient circulating PGT121-YTE to delay viral replication (blue line) for several days.

## Discussion

This study indicates that the induction of immune responses to immunotherapy in NHP may occur in two unrelated ways. Firstly, it is the first to show that the introduction of a triple YTE mutation in the Fc of the non-immunogenic PGT121 bnAb, designed to extend its PK profile and prolong protection against SHIV challenge when administered SC to macaques, unexpectedly rendered it immunogenic. Thus, to date, while nine out of eleven naïve macaques produced no anti-PGT121 antibody after one to three injections of the unmodified PGT121, ADA responses were observed in 7/10 naïve macaques that received PGT121-YTE at the same 5mg/kg dose (Table 2); such responses occurring following a single injection and enhanced by a second administration. The second means of induction of ADA, as evidenced by the two out of eleven macaques (# 11N006 and #JFL) that produced high levels of anti-id following each injection of unmodified PGT121 (Fig 1), appears to a direct consequence of pre-injection priming for specific anti-id production resulting in early (before day 7) and short lived anti-id responses. To date, these responses have been observed in 10–20% of macaques in the case of HIV bnAbs [24](Fig 1) in our laboratory.

**Table 2:** Summary of ADA induction and its outcome on pharmacokinetics and protection.

Experiment	Monkey #	Injection #1 ADABind^ nhib^^		Injection #2 ADA Bind Inhib		SHIV SF162P3 challenge	Comments, ADA types*	(Ref)/Fig
PGT121	5834	-		*-*	*-*		Non-immunogenic, no ant-id	(25)
	5844	-		*-*	*-*		Non-immunogenic, no anti-id	
PGT121	8291						Non-immunogenic, PK nor	(5)
	8338	-					Non-immunogenic, PK nor	
	8288	-					Non-immunogenic, PK nor	
	8390	-					Non-immunogenic, PK nor	
PGT121	5814*	-		*-*			Non-immunogenic, PK nor	
	5821	-		*-*			Non-immunogenic, PK nor	
PGT121	11N006	-		*-*		Protected	Non-immunogenic, no anti-id	Fig 1
	04N013	-		*-*		Protected	Non-immunogenic, no anti-id	
	07N008	-		*-*		Protected	Non-immunogenic, no anti-id	
	12N010	++	*+*	*++*	*++*	Protected	Treat-boosted anti-id	
	JFL	++	*++*	*+++*	*++*	Protected	Pre-existing anti-id	
PGT121-YTE	T769	+++	*+*	*+++*	*+++*		PK↓ #1, #2, Treat-boosted ADA	Fig 3
	T770	+/-	*+/-*	*+++*	*+*		PK↑ #1, PK↓ #2, Treat-induced ADA	
PGT121-YTE	12D010	+/-	*-*				PK ↑ ADA -ve	Fig 5
	09D181	+	*-*				PK ↑, ADA -ve	
	11D042	++	*+/-*				PK ↓, Treat-induced ADA?	
	12D046	++	*+/-*				PK ↓, Treat-induced ADA?	
PGT121-YTE	T765	++	*-*			Infected	Binding ADA ↑, VL ↑	Fig 7
	13D077	++	*-*			Infected	Binding ADA ↑, VL↑	
	T766	-	*-*			Protected	Binding ADA–ve, VL -ve	
	13D036	-	*-*			Protected	Binding ADA–ve, VL -ve	

* 5814 and #5821 were injected 3 times IV with PGT121 with normal PK profiles and no ADA.

^Binding ADA OD450: + = 0–0.5; ++ = 0.5–2.0; +++ = >2.

^^Inhibitory ADA ID50: + = 0–1/ 500; ++ = 1/500–1/2,000; +++ = >1/2,000.

VL = viral load, nor = normal, treat = treatment.

ADA responses were monitored using both a binding ELISA assay and an inhibition of neutralization assay to characterize the properties with the most clinical impact, initially the assumption being that the binding population would contain lower affinity and less functional subpopulations. In the pre-primed macaques in Fig 1, the binding ADA responses were induced in both macaques by day 7–8 after each injection with PGT121 and were anti-idiotypic in nature exhibiting reactivity only with PGT121 or its family member eg 10–1074 and not with VRC01 or 3BNC117. In contrast, unless preexisting levels were present in the macaques at the time of injection (#JFL), inhibitory anti-Id activity remained low (1/450) during primary responses, but could reach high titers after a second injection e.g. the ID50 of >1/17,000 in macaque #T769 (Fig 1).

The differences between titers of binding vs inhibitory anti-id was more apparent In the macaques injected with PGT121-YTE where only the binding ADA response to PGT121-YTE was functionally associated with more rapid clearance of the administered PGT121-YTE from the blood and subsequent lack of protection in challenged macaques. Although this discordance between titers of binding vs inhibitory ADA was not expected, it is known that the interactions between unmodified id and anti-id molecules predominantly occurs at the distal ends of the F(ab) arms (i.e. in the V domains) [38] and one speculation is that the inhibitory anti-id require binding to both arms of the bnAbs while binding anti-id requires the binding to a single Fab arm. In this case, the YTE-mediated alteration may have further negatively affected the geometry necessary for stable dimerization employing both arms [43]. Another possibility is that anti-idiotypic antibodies that block neutralization are directed against the antigen contact residues (CDRs), whilst anti-idiotypes that don’t block neutralization recognize the mutant framework residues in PGT121 heavy and light chains [44].

**Fig 8:**
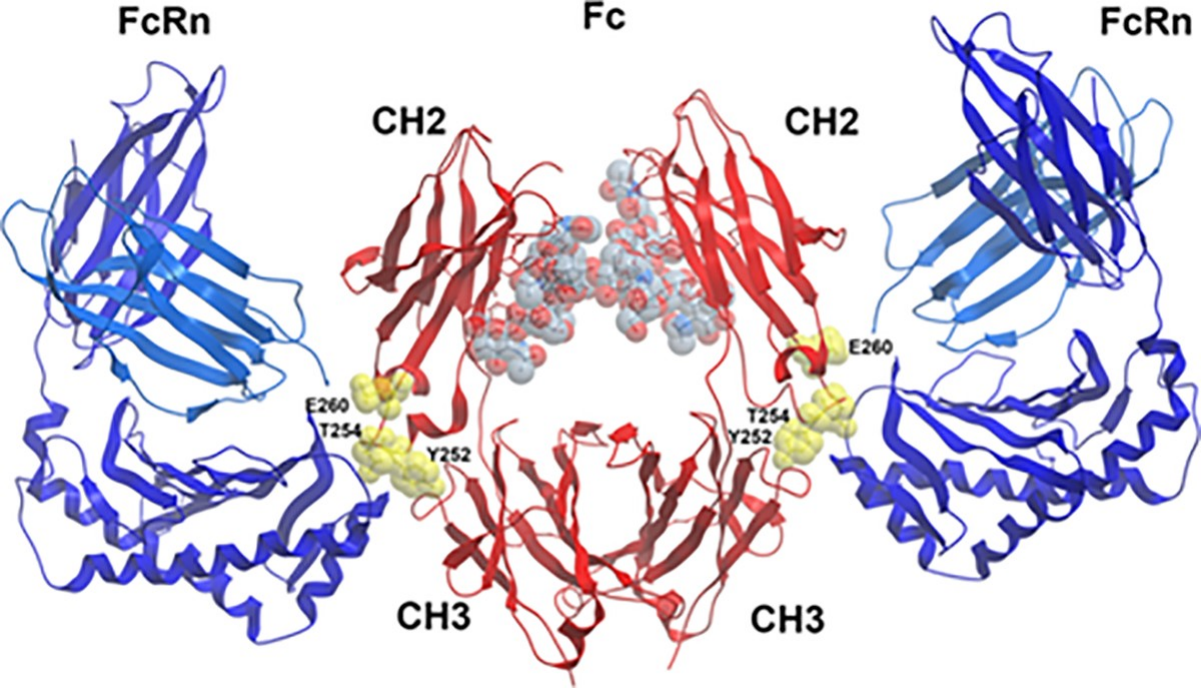
The structure of the YTE mutation in the context of FcRn binding is illustrated using PDB:4N08 originally published in by Oganesyan et al. [28]. The YTE mutation is at the CH2-CH3 interface and constitutes contact residues for FcRn binding. In its apo form, the YTE mutation is highly solvent exposed where it can constitute a discontinuous B cell epitope as suggested by the immunogenicty data shown in the manuscript.

There are several possibilities which may account for the induction of ADA by the PGT121-YTE mutant. The M252, S254 and T256 substitutions at the CH2/CH3 interface are in a hydrophobic “consensus” site that is highly exposed at the CH2/CH3 interface (Fig 8) and is recognized independently by Protein-A, Protein-G, the neonatal Fc-receptor and rheumatoid factor (RF) [45]. This consensus site is also recognized by peptides selected for Fc binding from phage-display libraries [45]. The non-polar nature of the consensus site enables the adaptive binding to each of these ligands, although the nature of this binding is distinct for each. In this regard, reactivity of the wild-type consensus sequence with RF shows that it can be immunogenic even in the autologous host [46].

In addition, hydrogen/deuterium exchange (HDX) analysis has indicated that the most notable difference between the binding of YTE and WT Fc to FcRn is the increased flexibility of the adjacent 244–254 segment of the CH2 domain, shown previously to correlate with decreased conformational stability. However surprisingly, distant segments in the VH, CH1 and VL domains also exhibited significantly increased flexibility in the YTE mutant; specifically linking IgG sites in both Fc and Fab regions to FcRn binding [47,48]. Thus, we postulate that the YTE mutations at the CH2/CH3 interface of a mAb leads to alterations in the Fc that render the molecule immunogenic due to the formation of neo T- or B-cell epitopes which “break” tolerance for the mAb, resulting in ADA responses specific for idiotopes in the Fab region, epitopes at the CH2/CH3 interface consensus site, or both.

Such YTE-dependent modifications would be expected to be present in the PGT121, PGDM1400 and 3BNC117 mutant bnAbs produced at PlantVax, since they all share the same IgG1 Fc genes which could explain the cross-reactivity observed between sera from anti-PGT121-YTE-injected macaques and PGDM1400-YTE and 3BNC117-YTE proteins in ELISA assays (Fig 6). These findings raise the possibility that if all of the HIV immunotherapeutic antibodies in a cocktail share the YTE mutation, cross-reactivity between the different bnAbs might result in immunogenicity and elimination, instead of overcoming the challenge of escape mutants.

In addition to the intrinsic differences between the mutant and WT PGT121, immunogenicity of PGT121-YTE appears to be also influenced by the previous environmental stimulation (both endogenous and exogenous) of the macaques. In this context, several definitions of ADA have been used to describe the immune responses following administration of therapeutic products [29] and translate well to the present study. Thus, the outcome of administering 5mg/kg of PGT121 and PGT121-YTE differed (i) in primed macaques with *pre-existing ADA* (#JFL), (ii) in primed animals with no pre-existing but with *treatment-boosted ADA* (#12N010,#T769) and (iii) in naïve animals which generate *treatment-induced ADA (#T770)* after a first or second injection. In addition, levels of *binding ADA*, whether boosted in primed animals or produced *de novo* in naïve macaques, were usually higher than *neutralization inhibition ADA* following a single injection except in one case, when the *pre-existing ADA* was detectable at the time of injection (Fig 1, #JFL). Table 2 summarizes the observed immune responses to PGT121 and PGT121-YTE injection/s in the 16 macaques described above in the context of these ADA types.

An FDA review has depicted temporal changes in ADA subpopulations in terms of Risk of Clinical Sequelae [49]. Thus, changes in binding ADA → PK altering ADA → neutralizing ADA → hypersensitivity ADA → cross-reactive neutralizing ADA were associated with increasing clinical severity and decreasing frequency. According to this scenario antibodies that impact function in vitro are more likely to be predictive of the clinical efficacy of such a treatment. However, using the current SHIV/macaque model, only ADA detected in binding assays showed a functional association with more rapid circulatory clearance (#T769, #T770, #12D010, #09D181) and lack of protection (#13D077, #T765), with pharmacokinetic profiles generally being the most sensitive means for evaluating the impact of immunogenicity. This finding is in agreement with Wang et al. [36] who reviewed the prescribing information of 121 biological products (43 mAbs, 26 enzymes, 11 cytokines, 12 growth factors and hormones and 29 peptides, proteins and toxins) which indicated that of the 108 products (89%) in which ADA (binding of anti-drug antibodies) was observed, 60% reported an immunogenicity impact on safety, 49% had an impact on efficacy and 26% an impact on PK with the latter being the most important metric in assessing immunogenicity.

In the case of humanized anti-RSV-YTE mAb (motavizumab, MEDI8897) safety studies showed the presence of low titres of ADA did not affect pharmacokinetics in adults [30]. In preterm infants with immature immune systems [31], post-baseline ADA was detected in 18 of 68 subjects (26.5%) between days 151 and 361 following IM injection and was considered likely to impact PK between these days. ADA titers detected reached 1:25,600 at the highest doses. This product is currently in an ongoing Phase IIB trial.

While it is well established that the induction of ADA may limit the efficacy of immunotherapeutic mAbs [36], the presence of anti-id in two of the 5 protected animals receiving SC PGT121 was therefore unexpected and highlighted both the importance of the immune status of the macaques and the timing of SHIV challenges. Macaques #12N010 and #JFL were both challenged fortuitously with SHIV SF162P3 at 24 hr after the first PGT121 administration before anti-id was induced and again after the second PGT121 injection at 8 weeks when anti-Id had returned to background levels (Fig 1) suggesting that (i) a non-immunogenic bnAb such as PGT121 can induce an anti-id response in macaques that are pre-primed and (ii) an injection of a single monoclonal bnAb into primed macaques may give rise to a truncated (3–4 weeks) anti-id immune response presumably as a result of complex formation and elimination of the idiotype.

While YTE-mutant mAbs are predicted to improve PK profiles following IV administration, there has been doubt as to their effect on bioavailability and efficacy when injected SC [50]. In this context, the YTE mutation did appear to increase circulatory retention by ~2-fold in macaques that did not produce high levels of ADA for two weeks after a single SC injection of PGT121-YTE (macaques #T770, #12D010, #09D181). It is anticipated however, that like #T770, a second injection would induce ADA and reduce plasma retention. It is not known whether the outcome of the YTE mutation on the non-immunogenic PGT121 is more consequential than other mAbs but it is possible, that since most unmodified human HIV bnAbs are highly mutated and induce ADA in naive macaques following a second injection or AAV-delivery [24,33,35], they may be particularly prone to YTE-mediated immune responses. Similar macaque protection studies with the PGT121-LS mutant are now ongoing to compare the YTE and LS mutations.

While the immunogenicity observed in macaques may not translate to humans, the recent HIV viral suppression study in humans using a combination 3BNC117 and 10–1074 immunotherapy, indicated that the duration of suppression ranged from 5 to >30 weeks in eleven trial recipients exhibiting complete viral suppression [51]. This variability is consistent with pre-priming or the production of ADA in some individuals and highlights the importance of employing optimal and sensitive ADA assays in human studies.
